# Imaging immune responses: visualizing immunity from molecules to medicine

**DOI:** 10.1038/s44303-026-00137-y

**Published:** 2026-02-02

**Authors:** I. Jolanda M. de Vries

**Affiliations:** Professor of Immunology, Head of Department Medical BioSciences, Radboud university medical center, Nijmegen, The Netherlands

## Abstract

Imaging technologies have become indispensable for understanding immune responses across molecular, cellular, and organism scales. By enabling real-time visualization of immune cell behaviour in health and disease, imaging bridges fundamental immunology and clinical application. This Editorial introduces the ESMI Collection, which showcases recent advances in imaging immune responses that support translational research and the development of precision medicine.

## The complexity of the immune system

The immune system is one of the most dynamic and intricate networks in the human body. It protects us from pathogens, repairs damaged tissue, orchestrates inflammation, and plays a decisive role in controlling tumours and determining the success of (immuno)therapies^1^. Comprising a diverse array of cells, organs, and molecular mediators, the immune system’s complexity has long challenged scientists seeking to study and observe its functions within living organisms. Understanding its dynamics by visualizing its components and behaviour in real time has therefore become one of the most powerful ways to uncover immunity in health and disease. Imaging provides an indispensable, yet challenging, window into this complexity.

## Imaging the immune system across different scales

Imaging immune activity combines molecular specificity with spatial and temporal resolution, offering an integrated view of how immune cells move, interact, and respond to their environment. It connects molecular insight with physiology, providing context to immunological mechanisms. The visualization of immune processes relies on an array of technical approaches that operate across molecular, cellular, tissue, and whole-body scales, each bringing unique strengths to this endeavour.

Optical methods using fluorescent or nanoparticle-based probes enable sensitive detection of immune markers that illuminate single cells or populations. Advances in intravital microscopy, including confocal or multiphoton approaches, now permit direct observation of immune cell dynamics within intact tissues of living organisms^2^. Complementary non-optical approaches, such as magnetic resonance imaging (MRI), computed tomography (CT), positron emission tomography (PET), and single-photon emission computed tomography, extend these observations to the organ and body level^3,4^. When combined, these tools provide a continuum of information, from molecular signalling events to systemic immune responses, bridging the gap between basic immunology and clinical application.

## Unravelling immunity in health and disease

By revealing when, where, and how immune reactions are initiated, regulated, and resolved, imaging deepens our understanding of normal immune surveillance and pathological responses, while informing the optimization of therapeutic strategies. In cancer research, for example, immuno-PET has been used to visualize effector T cells within tumours, providing a direct measure of immune activity and suppression^5,6^. Such studies are reshaping our understanding of intratumoral dynamics and enabling prediction of therapy responses, patient stratification, and early detection of resistance. Imaging of viral infections similarly sheds light on the immune dynamics during pathogen control, guiding the design of vaccines^7^.

By capturing immune activity as it unfolds, imaging connects the molecular to the macroscopic and the experimental to the clinical. It reveals the spatial and temporal patterns that underlie protection, pathology, and therapeutic success. In this sense, imaging the immune system is not only a tool, but also lays the groundwork for precision medicine.

## The ESMI collection: a platform for translation and innovation

To advance this field, the European Society for Molecular Imaging (ESMI) and *npj Imaging* have joined forces to launch the first-ever ESMI Collection, *Imaging Immune Responses*, highlighting state-of-the-art research at the intersection of imaging science, immunology, and clinical translation. This Collection marks the beginning of a broader partnership between ESMI and *npj Imaging*, within which multiple ESMI Collections will be developed, each addressing distinct topics of relevance to the ESMI community. Founded to advance molecular imaging across biological and medical research, the ESMI serves as an international scientific society connecting researchers, clinicians, and industry partners. ESMI fosters innovation and knowledge exchange through its annual meetings, educational initiatives, and support of early-career scientists, while promoting the development and translation of imaging technologies that address fundamental biological questions and clinical challenges.

Across this Collection series, the ESMI–*npj Imaging* partnership seeks to advance imaging technologies, analytical methodologies, and clinical translation, strengthening the dialogue between fundamental discovery and real-world implementation. By bringing together imaging scientists, life scientists, data scientists, engineers, and clinicians, this initiative aims to accelerate the responsible translation of imaging innovations into tangible clinical benefit and broader societal impact.

With the publication of this first ESMI Collection, we bring together research focused on advancing the imaging of immune function. We invite the imaging and biomedical research communities to engage with the work presented here and to consider contributing to this and future ESMI Collections, which will continue to highlight emerging themes and innovations across molecular and biomedical imaging. Through these Collections, we welcome ongoing submissions that help shape the scientific dialogue and advance the impact of imaging research.
